# Comparison of Bayesian and frequentist group-sequential clinical trial designs

**DOI:** 10.1186/s12874-019-0892-8

**Published:** 2020-01-07

**Authors:** Nigel Stallard, Susan Todd, Elizabeth G. Ryan, Simon Gates

**Affiliations:** 1 Statistics and Epidemiology, Division of Health Sciences, Warwick Medical School, University of Warwick, Coventry, UK; 2 Department of Mathematics and Statistics, University of Reading, Reading, UK; 3 Cancer Research UK Clinical Trials Unit, Institute of Cancer and Genomic Sciences, University of Birmingham, Birmingham, UK

**Keywords:** Adaptive design, Interim analysis, Type I error rate, Sequential analysis, Sequential design

## Abstract

**Background:**

There is a growing interest in the use of Bayesian adaptive designs in late-phase clinical trials. This includes the use of stopping rules based on Bayesian analyses in which the frequentist type I error rate is controlled as in frequentist group-sequential designs.

**Methods:**

This paper presents a practical comparison of Bayesian and frequentist group-sequential tests. Focussing on the setting in which data can be summarised by normally distributed test statistics, we evaluate and compare boundary values and operating characteristics.

**Results:**

Although Bayesian and frequentist group-sequential approaches are based on fundamentally different paradigms, in a single arm trial or two-arm comparative trial with a prior distribution specified for the treatment difference, Bayesian and frequentist group-sequential tests can have identical stopping rules if particular critical values with which the posterior probability is compared or particular spending function values are chosen. If the Bayesian critical values at different looks are restricted to be equal, O’Brien and Fleming’s design corresponds to a Bayesian design with an exceptionally informative negative prior, Pocock’s design to a Bayesian design with a non-informative prior and frequentist designs with a linear alpha spending function are very similar to Bayesian designs with slightly informative priors.This contrasts with the setting of a comparative trial with independent prior distributions specified for treatment effects in different groups. In this case Bayesian and frequentist group-sequential tests cannot have the same stopping rule as the Bayesian stopping rule depends on the observed means in the two groups and not just on their difference. In this setting the Bayesian test can only be guaranteed to control the type I error for a specified range of values of the control group treatment effect.

**Conclusions:**

Comparison of frequentist and Bayesian designs can encourage careful thought about design parameters and help to ensure appropriate design choices are made.

## Background

An increasing desire for efficiency in clinical trials has led to growing interest in adaptive designs. Frequentist group-sequential designs enable interim analyses to be performed during the conduct of a clinical trial without inflation of the overall type I error rate [1]. With an increased application of Bayesian methods in clinical trials, a number of researchers have proposed Bayesian group sequential methods [2, 3].

Not all proponents of Bayesian sequential designs consider exact control of the type I error rate essential [4]. Some, however, have suggested that the stopping rules for Bayesian group sequential designs should also be chosen in such a way that the frequentist type I error rate is controlled [2, 5, 6], particularly in the setting of phase III or late phase II clinical trials, when it is often considered desirable to control the risk of a false positive result, that is an erroneous conclusion that a new treatment is efficacious.

There are a number of published examples of trials using a Bayesian stopping rule chosen to control the type I error rate. Hueber et al. [7] (see also [8] for additional statistical details) describe a Bayesian group-sequential trial comparing secukinumab with placebo for the treatment of Crohn’s disease. The outcome is the change in Crohn’s Disease Activity Index (CDAI), which was taken to be normally distributed. Prior distributions were specified separately for the placebo and secukinumab effects, with the former being informative and the latter non-informative. Analyses were planned after 30 and 60 patients, when the trial could be stopped if both (i) the posterior probability that secukinumab was superior to the placebo exceeded 95%, and (ii) there was at least a 50% posterior probability that the change in CDAI due to secukinumab was superior to that for placebo by at least fifty. The type I error rate for this design was calculated using the R package gsbDesign[9] and shown to be 1.2% if the change in CDAI due to placebo was as anticipated.

A Bayesian group-sequential trial with a binary primary outcome is described by Wilber et al. [10]. This randomised trial compared antiarrhythmic drug therapy with catheter ablation for the treatment of paroxysmal atrial fibrillation. The primary outcome was the observation of protocol-defined treatment failure. Analyses were planned after 150, 175, 200 and 230 patients, with a stopping rule based on the posterior probability of superiority of the experimental treatment over the control exceeding 98%, giving a type I error rate of 0.025.

The increasing use of Bayesian sequential designs that control the frequentist type I error rate has led to a growing body of work comparing Bayesian and frequentist group sequential trial methods [3, 5, 8, 11–14]. This paper adds to this work. In contrast to some authors who draw comparisons between underlying Bayesian and frequentist paradigms, our focus is a practical one, in which we compare Bayesian and frequentist group sequential tests in terms of their boundary values and operating characteristics. We consider specifically the setting of normally distributed data or test statistics. This facilitates comparison between Bayesian and frequentist group sequential methods as the latter have been largely developed in this setting.

We consider separately Bayesian designs in which a single treatment effect is considered, either in a single-arm trial or with a prior specified directly for the difference between experimental and control treatments, and in which treatment effects have independent prior distributions. In the one-parameter setting frequentist and Bayesian group-sequential designs can be identical if sufficient flexibility in choice of design parameters is allowed [12], and we show that frequentist and Bayesian group-sequential designs may be very similar for common choices of stopping rules. In the two-parameter setting we show that the frequentist and Bayesian designs cannot correspond, and show that in this case the Bayesian group-sequential designs can only control the type I error rate for specified values of the control group treatment effect.

## Methods

### Notation and problem formulation

#### Single arm trials with normally distributed data

Suppose we conduct a group-sequential single-arm clinical trial of some experimental treatment with up to *K* analyses of a single sample of normally distributed data with a cumulative total of *n*_*k*_ observations at look *k*,*k*=1,…,*K*.

At each look the data observed up to that point will be analysed and a decision made whether or not to continue to the next look. We will only consider stopping the trial for a positive result, that is for efficacy. Additional stopping for futility is considered in the “Discussion” section.

Denoting by *Y*_*i*_ the observed value for patient *i*, we will assume this is normally distributed with mean *θ* and known variance *σ*^2^. We wish to draw inference on *θ* and will assume that parameterisation is such that *θ*=0 corresponds to the experimental treatment being of equal efficacy to some specified reference value or standard treatment effect, with positive values of *θ* (and hence of *Y*_*i*_) indicative of superiority of the experimental treatment.

Let $\bar Y_{k} = \sum _{i=1}^{n_{k}} Y_{i}/n_{k}$ denote the mean value from the cumulative sample at look *k*. This is the sufficient statistic for *θ* at look *k*. It is helpful to write the distribution in terms of the inverse of the variance, known as the information, and set *I*_*k*_=*n*_*k*_/*σ*^2^. We then have $\bar Y_{1}, \ldots, \bar Y_{K}$ multivariate normal with

1 $$  \left (\begin{array}{c} \bar Y_{1}\\ \vdots \\ \bar Y_{K}\end{array} \right) \sim N \left (\left (\begin{array}{c} \theta\\ \vdots \\ \theta\end{array} \right), \left (\begin{array}{cccc} I_{1}^{-1} & I_{2}^{-1} & \cdots & I_{K}^{-1} \\ I_{2}^{-1} & I_{2}^{-1} & \cdots & I_{K}^{-1} \\ \vdots & \vdots & \ddots & \vdots\\ I_{K}^{-1} & I_{K}^{-1} & \cdots & I_{K}^{-1} \end{array} \right) \right)  $$

with a similar multivariate normal distribution for the standardised test statistics, $\bar Y_{1} \sqrt {I_{1}}, \ldots, \bar Y_{K} \sqrt {I_{K}}$.

In a frequentist setting, we will test the null hypothesis, *H*_0_:*θ*≤0 against the one-sided alternative, *θ*>0, concluding that the experimental treatment is superior to the standard if this null hypothesis is rejected. The test will be based on the observed values of $\bar Y_{1}, \ldots, \bar Y_{K}$, stopping and rejecting the null hypothesis at look *k* if $\bar Y_{k}$ is sufficiently large as described in more detail below.

In a Bayesian setting, inference will be based on the posterior distribution for *θ* given the observed data. Basing the likelihood on (1), a normal prior for *θ* is conjugate. Given prior distribution $\theta \sim N\left (\theta _{0}, I_{0}^{-1}\right)$ the posterior distribution for *θ* following observation of $\bar Y_{k}=\bar y_{k}$ at look *k* is given by

2 $$  \theta \mid \bar y_{k} \sim N \left (\frac{\theta_{0} I_{0} + \bar y_{k} I_{k}}{I_{0}+I_{k}}, \frac{1}{I_{0}+I_{k}} \right)  $$

(see [15] Section 5.2). If this posterior distribution is sufficiently indicative of a positive treatment effect the trial will be stopped with the conclusion that the experimental treatment is superior to the standard or reference value. More details are given below.

The value of *I*_0_ gives a measure of the prior information. In particular, letting *I*_0_ approach 0 gives a flat improper normal prior.

#### Single arm trials with non-normal data

For non-normal data, tests can be based on the assumed distributional form parameterised in terms of the treatment effect, which will again be denoted by *θ*. An analytic form of the posterior distribution may be available if a conjugate prior distribution is used.

Alternatively, in many cases, if *n*_1_,…,*n*_*K*_ are sufficiently large, we can obtain an estimate $\hat \theta _{k}$ for the treatment effect based on the data at look *k* with $\hat \theta _{1}, \ldots, \hat \theta _{K}$ approximately following the multivariate normal distribution (1) for some *I*_1_,…,*I*_*K*_. It is common to use this approximate distributional form in a frequentist group-sequential test [16], enabling use of these estimates in place of the single sample means and applying methods based on the normal distribution (1) even without normally distributed data, or with normal data when the variance cannot be assumed to be known.

An illustration in the setting of a single sample of binomial data is given below.

#### Comparative trials

Suppose now we have two groups; group 0, the control group and group 1, the experimental treatment group. Let *Y*_*ji*_ denote the response from patient *i* in group *j*, assumed to be normally distributed with known variance, with $Y_{{ji}} \sim N\left (\mu _{j}, \sigma _{j}^{2}\right), j = 0,1$. We wish to draw inference on the treatment difference given by *θ*=*μ*_1_−*μ*_0_. We will again assume larger values of *Y*_*ji*_ are preferable so that larger values of *θ* correspond to the superiority of the experimental treatment to the control treatment.

At analysis *k*, suppose that we have a total of *n*_*jk*_ observations from group *j*, and let $\bar Y_{{jk}} = \sum _{i=1}^{n_{{jk}}} Y_{{ji}}/n_{{jk}}, j = 0,1, k=1, \ldots, K$. Writing $I_{{jk}} = n_{{jk}}/\sigma _{j}^{2}$, we have $\bar Y_{j1}, \ldots, \bar Y_{{jK}}$ multivariate normal with $\bar Y_{{jk}} \sim N\left (\mu _{j}, I^{-1}_{{jk}}\right)$ and $\text {cov}(\bar Y_{{jk}}, \bar Y_{jk^{\prime }}) = I^{-1}_{jk^{\prime }}$ if *k*<*k*^′^.

A sufficient statistic for *θ* at look *k* is $D_{k} = \bar Y_{1k} - \bar Y_{0k}$, with *D*_1_,…,*D*_*K*_ following the multivariate normal distribution as in (1) with $I_{k} = \left (\sigma _{1}^{2}/n_{1k}+\sigma _{0}^{2}/n_{0k}\right)^{-1}$.

In a frequentist setting, we will test *H*_0_:*θ*≤0 against *θ*>0 based on the observed values of *D*_1_,…,*D*_*K*_, stopping and rejecting the null hypothesis at look *k*, concluding that the experimental treatment is superior to the control, if *D*_*k*_ is sufficiently large, as described in more detail below.

In a Bayesian setting, we may specify the prior distribution for the treatment effect in two ways. The first is to specify a prior distribution for the treatment difference, *θ*, directly. Suppose again that *θ* has a normal prior distribution with $\theta \sim N\left (\theta _{0}, I_{0}^{-1}\right)$. At look *k* the posterior distribution for *θ* given observed value *D*_*k*_=*d*_*k*_ is given by

3 $$  \theta \mid d_{k} \sim N \left (\frac{\theta_{0} I_{0} + d_{k} I_{k}}{I_{0}+I_{k}}, \frac{1}{I_{0}+I_{k}} \right).  $$

The alternative is to specify independent prior distributions for *μ*_0_ and *μ*_1_, update these separately to obtain posterior distributions for *μ*_0_ and *μ*_1_ and then use these to obtain a posterior distribution for *θ*. This approach is considered in detail below in the section entitled “Comparison of frequentist and Bayesian group-sequential approaches - two parameter case”.

For non-normal data, or when the variance cannot be assumed known, we often again have estimates of the treatment effect, $\hat \theta _{k}$, approximately normally distributed, so that the distributional form (1) can be used. As in the two-sample case with normally distributed data, in the Bayesian setting we can either specify a prior for *θ* directly or specify independent prior distributions for treatment effects in the two groups.

### Bayesian group-sequential approach

In a Bayesian sequential trial, inference at look *k* will be based on the posterior distribution for *θ* given in the single group case by (2), in the two sample case when a prior distribution is specified for *θ* directly by (3) and in the two sample case when prior distributions are given for *μ*_0_ and *μ*_1_ by the expression (10) given below.

A common approach is to stop the trial, concluding that the experimental treatment is superior to the control if the posterior probability that *θ* exceeds 0 given the observed data is sufficiently large. In detail, critical values, *p*_*k*_,*k*=1,…,*K*, will be specified and the trial will stop as soon as

4 $$  Pr(\theta > 0 \mid \text{data at look } {k}) \ge p_{k}.  $$

Considering stopping to conclude the experimental treatment is superior to the control to be equivalent to rejection of *H*_0_, the frequentist type I error rate of this Bayesian sequential procedure can be calculated by noting that *Pr*(*θ*>0∣data at look *k*) is a random variable since it depends on the observed data. Control of the type I error rate is thus achieved if

5 $$ {}Pr (Pr(\theta \!>\! 0 \mid \text{data at look } {k}) \ge p_{k} \text{ some}\ k \le K; \theta\,=\,0) = \alpha.  $$

It has been suggested that *p*_1_,…,*p*_*K*_ should be chosen to satisfy this condition [2].

A number of alternatives to the stopping criterion (4) above have also been proposed. For example, the trial might be stopped to declare the experimental treatment superior at look *k* if the posterior probability that *θ* exceeds some specified positive target value, or the predictive probability that the experimental treatment would be found superior if the trial continued to the final analysis, is sufficiently large [8, 17, 18].

Although, in general, different values for *p*_1_,…,*p*_*K*_ could be specified, often a common value *p*_1_=⋯=*p*_*K*_ is used [2], with this value chosen to satisfy (5). We will consider both the general and this specific case in the examples below.

In many settings the probability on the left hand side of (5) can most easily be calculated via simulation methods [2]. In the case of single- or two-sample normally distributed data considered here, since, for a specified prior distribution, the posterior probability (4) depends on $\bar Y_{k}$, it can be calculated analytically from the joint distribution (1), for example in R using the gsbDesign [9] or code available from the first author.

### Frequentist group-sequential approaches

In a frequentist setting, the null hypothesis, *H*_0_:*θ*≤0, will be rejected, and the trial stopped at look *k* if $\bar Y_{k} \sqrt {I_{k}} \ge u_{k}$ for some *u*_*k*_ in the single-sample case or if $D_{k} \sqrt {I_{k}}\ge u_{k}$ in the two sample case. As the forms of the joint distributions for $\bar Y_{1},\ldots, \bar Y_{K}$ and *D*_1_,…,*D*_*K*_ are identical, we will here consider only the single-sample case.

To control the type I error rate at some specified level *α*, it is required to choose *u*_1_,…,*u*_*K*_ with $ Pr(\bar Y_{k} \sqrt {I_{k}}\ge u_{k}, \text { some} k \le K; \theta) \le \alpha $ for all *θ*≤0. The form (1) means that this is satisfied if

6 $$  Pr(\bar Y_{k} \sqrt{I_{k}}\ge u_{k} \text{ some}\ k \le K; \theta=0) = \alpha.  $$

As the requirement (6) is insufficient to specify *u*_1_,…,*u*_*K*_, a number of approaches have been proposed as described in the next two subsections.

#### Pocock’s test and O’Brien and Fleming’s test

Pocock [19] and O’Brien and Fleming [20] propose methods with equally-spaced looks, that is, using the notation introduced above, with *I*_*k*_=*kI*_*K*_/*K*,*k*=1,…,*K*. O’Brien and Fleming suggest stopping if $\bar Y_{k} I_{k}$ exceeds some fixed value, that is taking $u_{k} = c/\sqrt {I_{k}}$. Pocock suggests stopping if the standardised difference $\bar Y_{k} I_{k}^{1/2}$ exceeds a fixed value, that is taking *u*_*k*_=*c*. In each case, the constant value for *c* is found so as to satisfy (6). These values are tabulated for certain *K* and *α* [19, 20], or can be obtained from a numerical search, noting that the probability in (6) can be expressed in terms of the multivariate normal distribution function which may be evaluated numerically, for example in R using function pmvnorm in the mvtnorm package [21].

#### Spending function approaches

Slud and Wei [22] suggest introducing greater flexibility to sequential designs that satisfy (6) by specifying the type I error rate “spent” at each look. In detail, they specify *α*_1_≤⋯≤*α*_*K*_=*α*, then obtain *u*_*k*_,*k*=1,…,*K*, such that the probability under the null hypothesis of stopping at or before look *k*, say at some look *k*^′^ with *k*^′^≤*k*, is equal to *α*_*k*_, that is

7 $$  Pr(\bar Y_{k^{\prime}} \sqrt{I_{k^{\prime}}} \ge u_{k^{\prime}} \text{ some}\ k^{\prime} \le k; \theta=0) = \alpha_{k}.  $$

This approach was extended by Lan and DeMets [23], who proposed that *α*_1_,…,*α*_*K*_ be given by a function *α*^∗^(*t*) of the information time, with *t* at look *k* equal to *I*_*k*_/*I*_*K*_ so that *α*_*k*_=*α*^∗^(*I*_*k*_/*I*_*K*_),*k*=1,…,*K*. For general choice of non-decreasing *α*^∗^ with *α*^∗^(0)=0 and *α*^∗^(1)=*α*, the approaches of Slud and Wei and Lan and DeMets are equivalent provided *I*_1_,…,*I*_*K*_ are specified in advance. By defining the functional form of *α*^∗^, the Lan and DeMets approach enables calculation of *u*_1_,…,*u*_*K*_ to satisfy (6) when *I*_1_,…,*I*_*K*_ are not given in advance, providing they are independent of $\bar Y_{1}, \ldots, \bar Y_{K}$.

Lan and DeMets give forms for the spending function *α*^∗^(*t*) corresponding approximately to the Pocock test, with *α*^∗^(*t*)=*α* log(1+(*e*−1)*t*), and the O’Brien and Fleming test, with $\alpha ^{*}(t) = 2(1 - \Phi (z_{\alpha }/\sqrt {t})),$ where *Φ* denotes the distribution function for a standard normal and *z*_*α*_ denotes *Φ*^−1^(1−*α*), the upper 100*α* percentile of the standard normal distribution. Exact spending functions for these tests for a given number of looks can be obtained numerically from the joint distribution (1) [24]. Alternative spending function forms have been suggested [1, 25], including as a special case the linear spending function *α*^∗^(*t*)=*αt*.

The stopping boundary values *u*_1_,…,*u*_*K*_ may be computed recursively[1]; at look *k*, supposing *u*_1_,…,*u*_*k*−1_ and *I*_1_,…,*I*_*k*_ are known, we can use the joint distribution of $\bar Y_{1}, \ldots, \bar Y_{K}$ for *θ*=0 from (1) along with a numerical search to find *u*_*k*_ to satisfy (7). These calculations can be performed in R using the gsBound in the gsDesign package [26] or code available from the first author.

### Examples

To compare the Bayesian and frequentist group-sequential methods, we illustrate the two approaches using three simplified examples. These are described below.

#### Example 1: Single-arm trial with normally distributed data

Consider a single-arm trial with the outcome for patient *i* equal to *Y*_*i*_ with *Y*_*i*_∼*N*(*θ*,*σ*^2^) for some known *σ*. Suppose that *θ*=0 corresponds to a null value and *θ*=1 to a worthwhile treatment effect. We will assume that the trial is conducted in up to five stages, that is *K*=5, with these of equal size so that the number of patients included in the first *k* stages is *n*_*k*_=*nk*/*K*. We will further assume that *n*_*K*_=10*σ*^2^. With this sample size a fixed sample size trial with a hypothesis test conducted at a two-sided 5% level would have power of approximately 90%. This gives *I*_1_,…,*I*_5_=2,…,10.

**Fig. 1:**
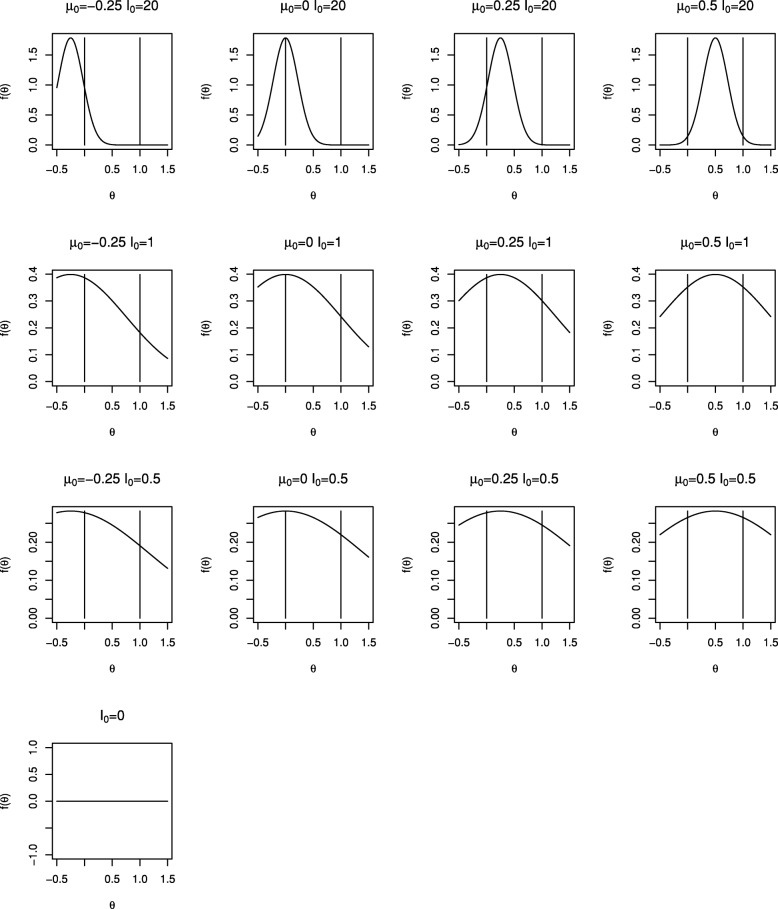
Densities for range of prior distributions for Bayesian sequential designs for Example 1

We will consider a range of prior distributions for *θ*. We will take *I*_0_ equal to 0 (non-informative), 0.5 and 1 (that is with weight equivalent to one twentieth and one tenth of the total information available from the trial) as well as a very informative prior distribution with *I*_0_=20, and will take *θ*_0_ equal to −0.25, 0, 0.25 and 0.5, recalling that 0 and 1 correspond to null and worthwhile treatment effects. Density functions for the range of prior distributions considered are shown in Fig. 1. The prior mean, *θ*_0_, increases across the columns moving from left to right and the prior information, *I*_0_, decreases as we move down the rows. The vertical lines correspond to the null and worthwhile treatment effects of 0 and 1. Only one plot is given in the lowest row as when *I*_0_=0 the prior distribution does not depend on *θ*_0_.

#### Example 2: Single-arm trial with binary data

Consider, as a second example, a single-arm trial with a binary outcome corresponding to success or failure for each patient. Suppose that the trial has up to four looks with 25, 50, 75 and 100 patients and assume that we wish to determine whether the true success rate, which will be denoted by *π*, exceeds a control rate, *π*_0_, assumed to be 0.5, using a non-informative prior distribution for *π*.

#### Example 3: Two-arm trial with normally distributed data

The third example is a two-arm trial with up to five equally-sized stages with the outcome for patient *i* in group *j* (*j*=0,1) equal to *Y*_*ij*_ with $Y_{{ij}} \sim N\left (\mu _{j}, \sigma _{j}^{2}\right)$ for some known *σ*_*j*_, where we assume *σ*_1_=*σ*_0_.

Denoting the treatment difference *μ*_1_−*μ*_0_ by *θ*, we will, as in Example Example 1: Single-arm trial with normally distributed data above, assume that *θ*=1 represents a worthwhile treatment effect. Assuming at stage *k* we have included a total of *n*_*k*_ patients in each of the two trial arms, we will set *I*_*k*_=*n*_*k*_/2*σ*^2^ and, again as in Example Example 1: Single-arm trial with normally distributed data, take *I*_1_,…,*I*_5_=2,…,10.

Suppose that *μ*_1_ and *μ*_0_ have independent normal prior distributions with $\mu _{j} \sim N\left (\mu _{j0}, I_{j0}^{-1}\right)$, with a moderately informative prior distribution for *μ*_0_ with *μ*_00_=0 and *I*_00_=0.5, and a noninformative prior distribution for *μ*_1_ with *I*_10_=0. The treatment difference *θ* thus has a non-informative prior distribution with *I*_0_=0.

## Results

### Comparison of frequentist and Bayesian group-sequential approaches - single parameter case

In this section we consider the setting in which we either have a single sample or are comparing two groups but specify a prior distribution for the treatment effect, *θ*, directly rather than giving separate prior distributions for *μ*_1_ and *μ*_0_. As noted above, in this case the two-sample setting is essentially identical to the single-sample settings, so that we will consider only the latter specifically.

Suppose that the maximum number of looks, *K*, the information at these looks, *I*_1_,…,*I*_*K*_ and, for the Bayesian design, the prior distribution parameters, *θ*_0_ and *I*_0_ are specified.

The posterior distribution for *θ* at look *k* in this case is given by (2) so that the posterior probability that *θ* exceeds 0 is given by

8 $$  Pr(\theta > 0 \mid \bar y_{k}, I_{k}) = 1 - \Phi \left (\frac{-\bar y_{k} I_{k} - \theta_{0} I_{0}}{\sqrt{I_{0}+I_{k}}} \right).  $$

Given some choice of *p*_1_,…,*p*_*K*_, for the Bayesian design using stopping criterion (4) expression (8) means that the trial will be stopped at look *k* if $\bar Y_{k} \sqrt {I_{k}} \ge u^{B}_{k}$ where

9 $$  u^{B}_{k} = \frac{-\theta_{0} I_{0} - \sqrt{I_{0}+I_{k}} \Phi^{-1}(1-p_{k})}{\sqrt{I_{k}}}  $$

so that the Bayesian trial, like the frequentist one, will stop whenever $\bar Y_{k}$, or equivalently the standardised $\bar Y_{k} \sqrt {I_{k}}$, is sufficiently large.

#### Sequential tests with general *α*_1_,…,*α*_*K*_ or *p*_1_,…,*p*_*K*_

With $u^{B}_{k}$ as given by (9), let $ \alpha ^{B}_{k} = Pr(\bar Y_{k^{\prime }} \sqrt {I_{k}}\ge u^{B}_{k^{\prime }} \text { some} k^{\prime } \le k; \theta =0). $ This may be calculated from the multivariate normal distribution of $\bar Y_{1} \sqrt {I_{1}}, \ldots, \bar Y_{K} \sqrt {I_{K}}$ following from (1). Setting *k*=*K* enables analytic calculation of the frequentist type I error rate for the Bayesian test.

Setting $\alpha _{k} = \alpha ^{B}_{k}$ and constructing a frequentist design using these *α*_1_,…,*α*_*K*_ values will give a frequentist group-sequential boundary identical to the Bayesian one.

Similarly, given frequentist group sequential spending function values *α*_1_,…,*α*_*K*_, we can obtain *u*_1_,…,*u*_*K*_ to satisfy (7). A Bayesian design with $p_{k} = 1 - \Phi \left ((-u_{k} \sqrt {I_{k}} - \theta _{0} I_{0})/\sqrt {I_{0}+I_{k}} \right), k=1, \ldots, K$, will then be identical to this frequentist one.

Thus, as noted by Emerson et al. [12], if we allow full flexibility over the choice of *p*_1_,…,*p*_*K*_ for the Bayesian group-sequential design and *α*_1_,…,*α*_*K*_ for the frequentist design, subject respectively to the constraint on overall type I error rate (5) or (6), the classes of frequentist group sequential and Bayesian designs are identical.

Similarly, if Bayesian sequential boundaries are constructed using the posterior probability that *θ* exceeds a positive target value or the posterior predictive probability of a final positive result, the fact that both of these are monotonically increasing in $\bar Y_{k}$ means that the stopping boundaries are again of the form $\bar Y_{k} \sqrt {I_{k}}\ge u^{B}_{k}$ for some $u^{B}_{1}, \ldots, u^{B}_{K}$, so that these still correspond to a frequentist boundary for appropriate choice of *α*_1_,…,*α*_*K*_ and vice versa [12]. The same result holds for sequential tests based on Bayes factors provided these are constructed so as to be monotonically increasing in $\bar Y_{k}$, as is the case, for example, when a point null at *θ*=0 is compared to a ‘one-sided’ prior with support for positive *θ* only.

#### Specific group-sequential tests: Single-arm trial with normally distributed data

Although in principle, *p*_1_,…,*p*_*K*_ and *α*_1_,…,*α*_*K*_ may be chosen arbitrarily, in practice, constraints may be put on the values used. In this case frequentist and Bayesian group sequential tests may not correspond. In this section we construct frequentist group-sequential designs with a linear alpha spending function and with alpha spending functions corresponding to the Pocock design and the O’Brien and Fleming design, comparing these with Bayesian tests with stopping criteria given by (4) with *p*_1_=⋯=*p*_*K*_.

Consider Example Example 1: Single-arm trial with normally distributed data above with the range of prior distributions illustrated in Fig. 1. In each case we used stopping criterion (4) and took *p*_1_=⋯=*p*_*K*_, finding the common value to give overall type I error rate of *α*=0.025.

**Fig. 2:**
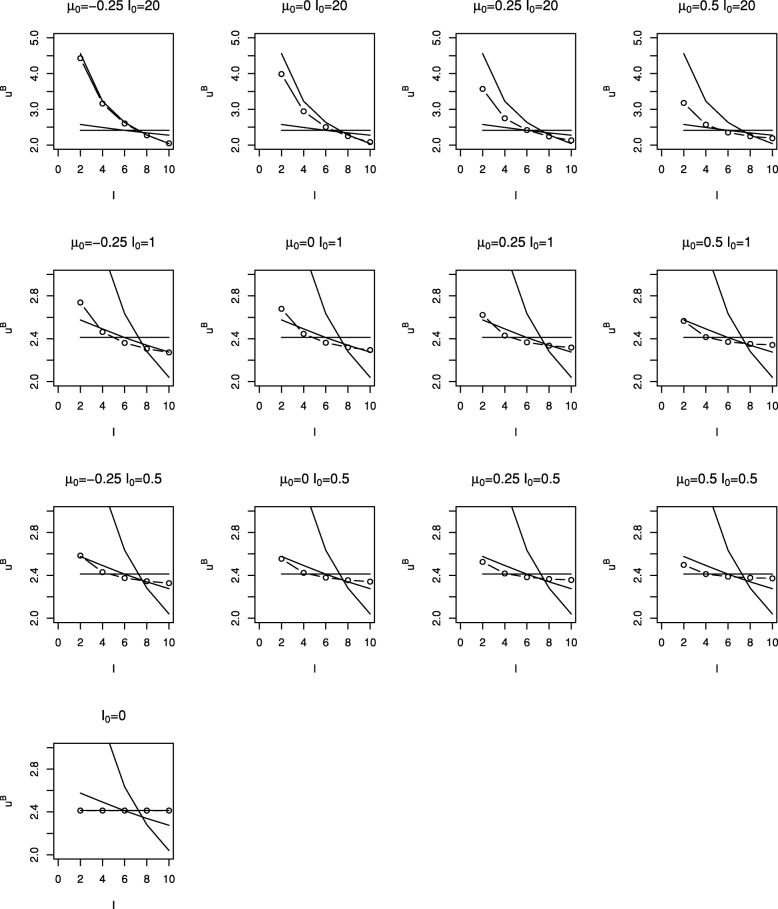
Stopping boundaries for Bayesian sequential tests with 5 looks using prior distributions from Figure 1 (∘). Solid lines give boundaries for O’Brien and Fleming test (steep sloping lines), Pocock test (horizontal lines) and for frequentist test with *α*^∗^(*t*)=*αt* (shallow sloping lines)

**Fig. 3:**
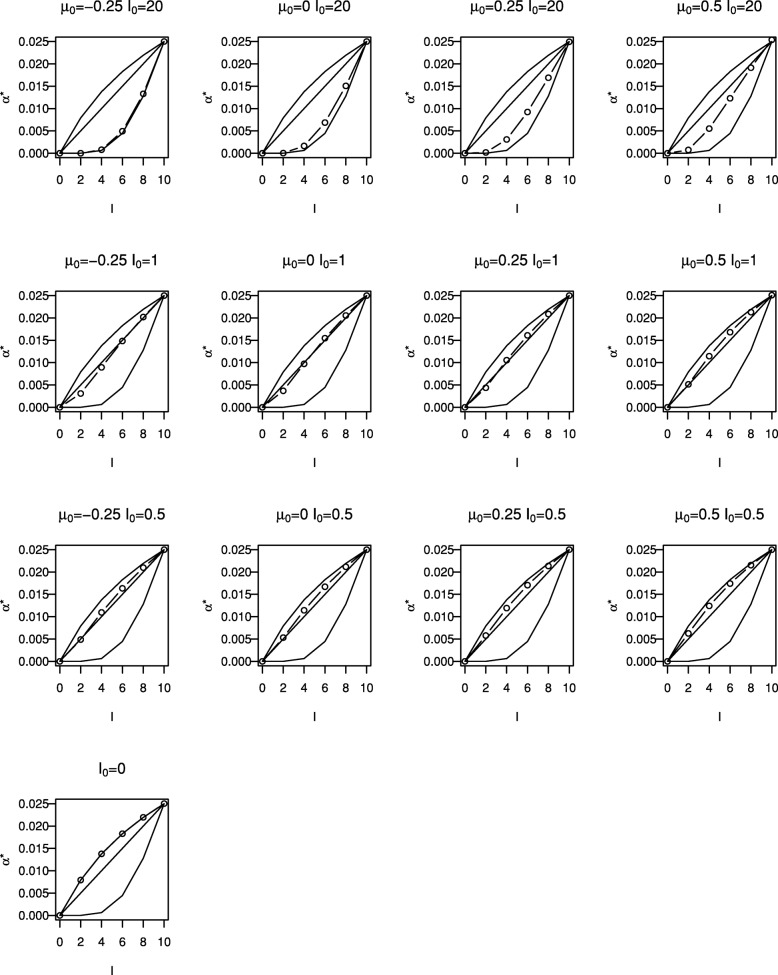
Cumulative type I error spent for Bayesian sequential tests shown in Fig. 2 (∘). Solid lines give boundaries for O’Brien and Fleming test (lower line), Pocock test (upper line) and for frequentist test with *α*^∗^(*t*)=*αt* (middle line)

Figure 2 shows critical values, $u^{B}_{1}, \ldots, u^{B}_{5}$, (plotted as circles) for the Bayesian tests with different prior distributions. Each plot corresponds to a different prior distribution, the layout of plots in the figure matching those in Fig. 1. Note that a different scale is used for the plots in the uppermost row. Using a similar format, Fig. 3 shows the cumulative type I error spent by each look for the tests shown in Fig. 2. Critical values and cumulative type I error spent are also given in Table 1.

**Table 1:** Boundary values and type I error rate spent for Bayesian and frequentist five-look group sequential tests

Bayesian tests				
*I*_0_	*θ*_0_	*p*_1_=⋯=*p*_5_	$u^{B}_{1}, \ldots, u^{B}_{5}$	$\alpha ^{B}_{1}, \ldots, \alpha ^{B}_{5}$
20.0	-0.25	0.6063	4.43, 3.16, 2.60, 2.27, 2.05	0.0000, 0.0008, 0.0049, 0.0133, 0.0250
1.0	-0.25	0.9818	2.74, 2.46, 2.36, 2.31, 2.27	0.0031, 0.0089, 0.0148, 0.0202, 0.0250
1.0	0.00	0.9856	2.68, 2.45, 2.36, 2.32, 2.29	0.0037, 0.0097, 0.0155, 0.0205, 0.0250
1.0	0.25	0.9889	2.62, 2.43, 2.37, 2.34, 2.32	0.0044, 0.0105, 0.0161, 0.0209, 0.0250
1.0	0.50	0.9914	2.57, 2.42, 2.37, 2.35, 2.34	0.0051, 0.0114, 0.0168, 0.0213, 0.0251
0.5	-0.25	0.9872	2.58, 2.43, 2.37, 2.35, 2.33	0.0049, 0.0110, 0.0163, 0.0210, 0.0250
0.5	0.00	0.9888	2.55, 2.42, 2.38, 2.36, 2.34	0.0053, 0.0114, 0.0167, 0.0212, 0.0250
0.5	0.25	0.9903	2.53, 2.42, 2.38, 2.37, 2.36	0.0058, 0.0119, 0.0171, 0.0213, 0.0250
0.5	0.50	0.9916	2.50, 2.41, 2.39, 2.38, 2.37	0.0063, 0.0124, 0.0174, 0.0215, 0.0250
0.0	0.00	0.9921	2.41, 2.41, 2.41, 2.41, 2.41	0.0079, 0.0138, 0.0183, 0.0220, 0.0250
Frequentist tests				
			*u*_1_,…,*u*_5_	*α*_1_,…,*α*_5_
O’Brien & Fleming			4.56, 3.23, 2.63, 2.28, 2.04	0.0000, 0.0006, 0.0045, 0.0128, 0.0250
Pocock			2.41, 2.41, 2.41, 2.41, 2.41	0.0079, 0.0138, 0.0183, 0.0219, 0.0250
*α*^∗^(*t*)=*αt*			2.58, 2.49, 2.41, 2.34, 2.28	0.0050, 0.0100, 0.0150, 0.0200, 0.0250

It can be seen that more informative or more negative priors lead to a smaller chance of stopping at earlier interim analyses; this makes sense as more information is required to overcome the prior and obtain a posterior probability $pr(\theta > 0 \mid \bar y_{k}) \ge p_{k}$. Other than for the most informative priors considered, it appears that the choice of *θ*_0_ has relatively little impact; in these cases the value of *I*_0_ is small relative to *I*_*K*_ so that the prior distribution makes relatively little contribution to the posterior distribution and hence to the stopping decision.

Figures 2 and 3 and Table 1 also show stopping boundaries and type I error spending functions for O’Brien and Fleming’s test, Pocock’s test and the frequentist test with a linear spending function, that is with *α*^∗^(*t*)=*αt*, for five equally-spaced analyses. Boundary values and type I error spent at each look for the different tests (omitting those with *I*_0_=20 and *θ*_0_>−0.025) are also given in Table 1, together with the value of *p*_1_=⋯=*p*_*K*_ required to give overall type I error rate of 0.025 for the Bayesian designs.

It can be seen that stopping boundaries and type I error spent for the O’Brien and Fleming test are nearly identical to those for the Bayesian test with prior distribution with *θ*_0_=−0.25 and *I*_0_=20. In this case the form of the stopping boundary, with stopping very unlikely at interim analyses but relatively likely at the final analysis, is only achieved if very strong negative prior opinion is held. This prior distribution was included specifically because of this similarity; it is hard to imagine anyone conducting a trial if they had such a strongly negative prior opinion of the effect of the treatment under investigation.

The similarity between Pocock’s test and the Bayesian test with a non-informative prior distribution for *θ* can also be noted. For a non-informative prior, that is with *I*_0_=0, (9) gives $u^{B}_{k} = -\Phi ^{-1}(1-p_{k})$ so that taking *p*_1_=⋯=*p*_*K*_ corresponds to taking $u^{B}_{1} = \cdots = u^{B}_{K}$. Thus in this case the Bayesian test with *p*_*k*_ chosen to control the overall error rate is identical to Pocock’s test when looks are equally spaced in terms of information.

For moderately informative prior distributions, that is for *I*_0_ equal to 0.5 or 1, the Bayesian test appears to be similar to the frequentist test with *α*^∗^(*t*)=*αt* for the reasonably wide range of *θ*_0_ values considered.

#### Specific group-sequential tests: Single-arm trial with binary data

Consider next Example Example 2: Single-arm trial with binary data above. In this case a Bayesian sequential test can be based on the exact binomial distribution of the data. In detail, denoting by *X*_*k*_ the number of successes observed from the *n*_*k*_ patients observed up to look *k*, *k*=1,…,4, we can take *X*_*k*_∼*Bin*(*n*_*k*_,*π*). A beta prior distribution is conjugate and a non-informative prior is *π*∼*Beta*(1,1), or equivalently *π*∼*U*[0,1]. The posterior distribution at look *k* after observing *X*_*k*_=*x*_*k*_ is then *π*∣*x*_*k*_,*n*_*k*_∼*Beta*(*x*_*k*_+1,*n*_*k*_−*x*_*k*_+1).

To be consistent with the notation above, where *θ* denotes the treatment effect with *θ*=0 corresponding to the null hypothesis, we can take *θ*=*π*−*π*_0_. The trial will stop to claim that *θ*>0, or equivalently, *π*>*π*_0_, if the posterior probability *Pr*(*π*>*π*_0_∣*x*_*k*_,*n*_*k*_)≥*p*_*k*_ for some *p*_*k*_.

Taking *p*_1_=⋯=*p*_*k*_, for a given value of *p*_1_, critical values in terms of the required number of successes at each look can be found by calculating this posterior probability for a range of possible *x*_*k*_ values. These in turn can be used to calculate the resulting frequentist type I error rate under the null hypothesis *H*_0_:*θ*=0 or equivalently in this case, *π*=*π*_0_=0.5, either by simulation or calculation and summation of the appropriate binomial probabilities. A numerical search can then be used to find the value of *p*_1_ at which the type I error rate is controlled at a specified level.

For a four-look test with a non-informative *Beta*(1,1) prior distribution for *π*, the type I error rate is controlled at level 0.05 for *p*_1_=⋯=*p*_4_=0.977. The critical values for the test in terms of the total number of successes observed at looks 1 to 4 are then respectively 18, 33, 47 and 61.

A frequentist group-sequential analysis can be based on the normal approximation (1) for $\hat \theta = X_{k}/n_{k} - \pi _{0}$ and $I_{k}^{-1} = \pi _{0} (1-\pi _{0})/n_{k}$. A four-look frequentist group-sequential Pocock test constructed based on this approximation would stop for $\hat \theta _{k} \sqrt {I_{k}} \ge u_{k}$ with *u*_*k*_=2.067, that is for $X_{k} \ge 0.5 n_{k} + 2.067 \sqrt {n_{k}}/2$, giving stopping boundaries in terms of *X*_*k*_ for *n*_*k*_=25,50,75 and 100 of 17.7, 32.3, 46.5 and 60.3. Rounding these up to integers gives stopping boundary values identical to those for the Bayesian test with a non-informative prior distribution.

#### Specific group-sequential tests: Two-arm trial with normally distributed data

We next consider Example Example 3: Two-arm trial with normally distributed data above, using only the prior information given by the prior distribution for the treatment difference *θ*, that is the non-informative prior distribution with *I*_0_=0.

The distribution of the observed difference between the treatment means at looks 1 to *K*, *D*_1_,…,*D*_*K*_ follows a multivariate normal distribution of the same form as that of the mean values $\bar Y_{1}, \ldots, \bar Y_{K}$ in the single-group case, with *I*_*k*_ now taken to be *n*_*k*_/2*σ*^2^. Setting *p*_1_=⋯=*p*_*K*_ and taking this value so as to control the overall type I error rate to be 0.025, thus gives critical values, *u*_*k*_, now for $D_{k} \sqrt {I_{k}}$, equal to 2.41 at all looks, exactly as in single-arm case with a non-informative prior distribution for *θ*.

### Comparison of frequentist and Bayesian group-sequential approaches - two parameter case

Consider now the setting in which we are comparing two groups of normally distributed data and, in the Bayesian setting, specify separate independent normal prior distributions for *μ*_1_ and *μ*_0_.

Suppose that the prior distributions are given by $\mu _{j} \sim N\left (\mu _{j0}, I_{j0}^{-1}\right), j=0,1$. Given observation of $\bar Y_{{jk}} = \bar y_{{jk}}$, the posterior distribution for *μ*_*j*_ is given by

$$\mu_{j} \mid \bar y_{{jk}} \sim N \left (\frac{\mu_{j0} I_{j0}+ \bar y_{{jk}} I_{{jk}}}{I_{j0}+I_{{jk}}}, \frac{1}{I_{j0}+I_{{jk}}} \right). $$

As *μ*_0_ and *μ*_1_ have independent prior distributions, their posterior distributions are also independent, so that the posterior distribution for *θ* is given by

10 $$ {\begin{aligned} \theta \mid \bar y_{1k}, \bar y_{0k}&\sim N \left (\frac{\mu_{10} I_{10}+ \bar y_{1k} I_{1k}}{I_{10}+I_{1k}} \right.\\&\left.- \frac{\mu_{00} I_{00}+ \bar y_{0k} I_{0k}}{I_{00}+I_{0k}}, \frac{1}{I_{10}+I_{1k}} + \frac{1}{I_{00}+I_{0k}}\right). \end{aligned}}  $$

Note that although in this case the prior distribution for *θ* is again normal, with *θ*∼*N*(*θ*_0_,*I*_0_) with *θ*_0_=*μ*_10_−*μ*_00_ and $I_{0}^{-1} = I_{10}^{-1} + I_{00}^{-1}$, the posterior distribution given by (10) is not generally the same as (3) that was obtained when the prior distribution for *θ* was considered directly.

It is shown in Appendix A that the posterior variance of *θ* when separate prior distributions are given for *μ*_1_ and *μ*_0_ given by (10) is always smaller than that given by (3) when only the prior distribution for *θ* is used. With independent prior distributions for *μ*_1_ and *μ*_0_, the posterior distribution depends on $\bar y_{1k}$ and $\bar y_{0k}$, and not just on the difference $D_{k} = \bar Y_{1k} - \bar Y_{0k}$. Assuming *μ*_1_ and *μ*_0_ are independent means that *θ* is not independent of *μ*_1_+*μ*_0_. Thus although *D*_*k*_ is sufficient for *θ*, we can also learn about *θ* by learning about *μ*_1_+*μ*_0_, for which *D*_*k*_ is not sufficient. We therefore gain information by knowing $\bar y_{1k}+ \bar y_{0k}$ as well as $\bar y_{1k}- \bar y_{0k}$, that is by having information on both $\bar y_{1k}$ and $\bar y_{0k}$, leading to a smaller posterior variance.

Suppose that, as in the single parameter case, we stop the trial as soon as we have *Pr*(*θ*>0∣data at look *k*)≥*p*_*k*_, and that we wish to choose *p*_1_,…,*p*_*K*_ so as to control the type I error rate to be at most *α*, that is to satisfy (5).

It is shown in Appendix B that, irrespective of the values of *p*_1_,…,*p*_*k*_, the stopping regions for frequentist and Bayesian group-sequential tests cannot coincide other than in the special case with *I*_1*k*_/(*I*_10_+*I*_1*k*_)=*I*_0*k*_/(*I*_00_+*I*_0*k*_),*k*=1,…,*K*, when the posterior distribution for *θ* is exactly the same as that obtained directly from a single prior distribution for *θ* without considering prior distributions for the means of the two groups separately,

With independent prior distributions for *μ*_1_ and *μ*_0_ the posterior distribution of *θ* depends on $\bar y_{1k}$ and $\bar y_{0k}$. The probability in (5) thus depends on *μ*_0_ and *μ*_1_ and the requirement that this is controlled at level *α* when *θ*=0 requires that it is controlled when *μ*_1_=*μ*_0_ for all values of *μ*_0_. Appendix B shows that beacuse the mean of the posterior distribution for *θ* when *μ*_1_=*μ*_0_ depends on *μ*_0_, this is impossible.

For the two-arm Bayesian group-sequential trial with five looks in Example Example 3: Two-arm trial with normally distributed data above, controlling the one-sided type I error rate to be 0.025 when *μ*_1_=*μ*_0_=0 requires *p*_1_=⋯=*p*_5_=0.9884.

**Fig. 4:**
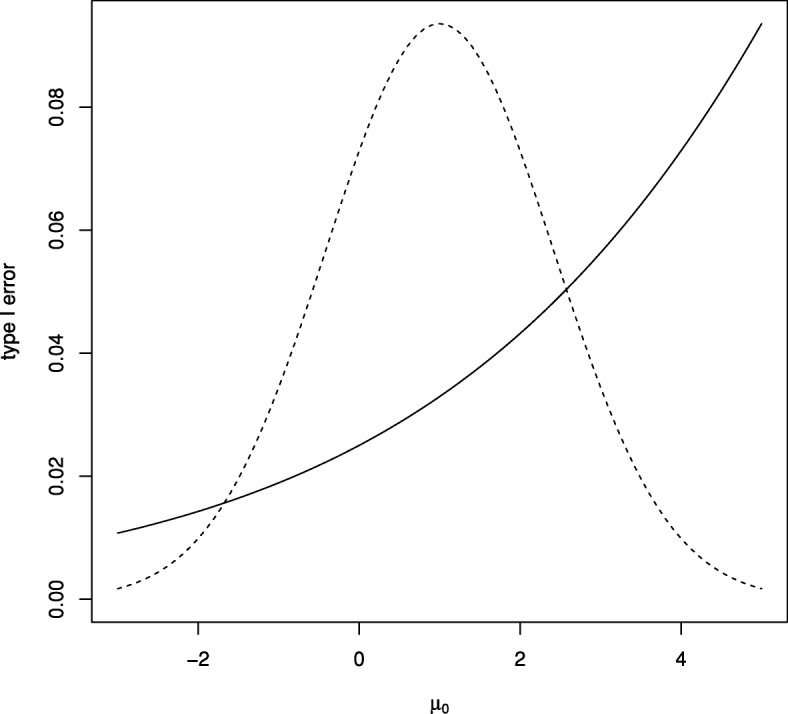
Type I error rate for Bayesian test with *K*=5 and *p*_1_=⋯=*p*_5_=0.9884 for range of true *μ*_0_ values along with density (not to scale) for the prior distribution for *μ*_0_

Figure 4 shows the one-sided type I error rate for this design for a range of *μ*_0_ values with, in each case, *μ*_1_=*μ*_0_ so that *θ*=0. It can be seen that in this case although the type I error rate is controlled for *μ*_0_=0, the type I error rate increases above the desired level for *μ*_0_>0. The figure also shows the prior distribution for *μ*_0_, showing that error rate inflation would occur for plausible values of *μ*_0_.

## Discussion

Our comparison has been restricted on the whole to group-sequential tests based on normally distributed test statistics. Although some exact or non-normal frequentist group-sequential test methods have been proposed [27–29] the assumption of normality is common in this setting. In Bayesian group-sequential tests it is more common to use non-normal distributions, with simulation methods being used if necessary to calculate operating characteristics. The decision to focus on normally distributed test statistics was made so as to put Bayesian and frequentist designs in a similar setting, facilitate comparison and identify relationships, such as that between the Pocock test and the Bayesian test with a non-informative prior distribution, which might otherwise not be apparent. As can be seen from the binary data example above, where the Pocock test and the exact Bayesian test give identical stopping rules, in practice asymptotic normality can be a reasonable assumption.

We have considered stopping for a positive result only. In practice, with both frequentist and Bayesian group-sequential designs, it is often desirable to allow stopping when a lack of efficacy is clear, that is for futility. Futility stopping rules can be divided into those that are binding, when the rule is specified in advance and must be adhered to in order to maintain the required properties of the design, and those that are non-binding, where a more flexible approach can be taken. As stopping for futility cannot lead to a positive claim of efficacy, it can only decrease the type I error rate. Thus with a non-binding futility stopping rule, it is desirable to control the type I error rate even if no futility stopping occurs, that is in the case when the trial is only stopped for a positive result as considered above. The use of a binding futility stopping rule will change the operating characteristics of the group-sequential tests.

We have focussed on comparison of Bayesian and frequentist group-sequential designs for single-arm and comparative studies. These are just one type of adaptive design, which can include many other features including adaptive exploration of a dose-response relationship, adaptive randomisation, dropping of arms in multi-arm trials, incorporation of multiple endpoints and sample size reestimation. Frequentist methods that guarantee control of error rates are available for some of these problems such as sample size re-estimation [30] but in some other cases construction of decision rules for frequentist methods can be challenging. Bayesian methods can be accompanied by simulations to verify operating characteristics under a likely range of scenarios for a wide variety of adaptations for which rigorous proof of error rate control is not available.

## Conclusions

Although Bayesian and frequentist group-sequential approaches are based on fundamentally different paradigms, in practice, when used for the analysis of a clinical trial, both provide an indication of the efficacy of an experimental treatment. This means that a comparison of Bayesian and frequentist test can be helpful to understand the frequentist operating characteristics for Bayesian tests and the Bayesian model and prior distributional assumptions that could lead to a particular frequentist test. This has been our aim in this paper.

Focussing on a setting in which test statistics can be assumed to be normally distributed, we have shown that in comparative trials with independent prior distributions specified for treatment effects in different groups, stopping rules from Bayesian and frequentist group-sequential designs cannot generally correspond. In this case the Bayesian group-sequential design can then only control the type I error rate for specified values of the control group treatment effect. Conversely, in single-arm trials, or when a prior distribution is specified for the treatment difference, stopping rules for Bayesian and frequentist group-sequential tests can be identical if full flexibility for both classes of designs is allowed, or can closely correspond for common choices of design parameters.

O’Brien and Fleming’s design was found to correspond closely to a Bayesian design with an exceptionally informative negative prior, this prior leading to the very small probability of early stopping for this design. The fact that such a prior is unlikely to represent prior belief suggests that the use of this design might not be appropriate without very careful thought.

In a similar way, noting that the Bayesian design with a non-informative prior and *p*_1_=⋯=*p*_*K*_ corresponds to a Pocock design suggests that this might also not be generally appropriate given the criticism that this design gives too high a probability of early stopping [31]. This illustrates the importance of appropriate choice of a prior distribution, rather than the general use of a non-informative prior. Evaluation of the frequentist properties can be useful in understanding the influence of the prior distribution in a Bayesian group-sequential design in which the overall type I error rate is controlled.

Bayesian adaptive methods are often more bespoke than frequentist approaches, with simulations used to evaluate their performance not only for a range of treatment effect scenarios but also allowing for anticipated data patterns arising from, for example, delayed responses, multiple endpoints including early outcomes, or different recruitment and drop-out rates. This can require more design work than the use of a more standard frequentist method but can be advantageous in that design choices and their consequences are considered carefully. It is recommended that if frequentist methods are used, equal care should be taken over design choices and their properties explored, using simulations if necessary.

## Appendix A: Comparison of posterior variances for comparative trials with single or independent prior distributions

Suppose we are in the two-group setting and have independent prior distributions with $\mu _{j} \sim N\left (\mu _{j0}, I_{j0}^{-1}\right), j=0,1$ and that we have observation of $\bar Y_{{jk}}$ with $\bar Y_{{jk}} \sim N(\theta, I^{-1}_{k}), j = 0,1, k = 1, \ldots, K$, so that the posterior distribution for *θ* is given by (10).

Considering only the single parameter *θ*, the posterior distribution is given by (10) with $\theta _{0} = \mu _{10} - \mu _{00}, I_{0}^{-1} = I_{00}^{-1} + I_{10}^{-1}$ and $I_{k} = \left (I_{0k}^{-1}+I_{1k}^{-1}\right)^{-1}$.

Let *I*_[1]*k*_ and *I*_[2]*k*_ denote the inverses of the posterior variance for *θ* in the one-parameter and two-parameter cases respectively. We will show that *I*_[1]*k*_≤*I*_[2]*k*_.

We will denote by *r*_0_ the ratio *I*_10_/*I*_00_, so that *I*_10_=*r*_0_*I*_00_, by *r*_*k*_ the ratio *I*_1*k*_/*I*_0*k*_, and by *Λ*_*k*_ the ratio *r*_*k*_/*r*_0_ so that *r*_*k*_=*Λ*_*k*_*r*_0_ and *I*_1*k*_=*Λ*_*k*_*r*_0_*I*_0*k*_. Without loss of generality, we will take *I*_0*k*_=1 so that *I*_1*k*_=*Λ*_*k*_*r*_0_. We then have $I_{[1]k} = I_{00}/\left (1 + r_{0}^{-1}\right) + 1/\left (1+(\Lambda _{k} r_{0})^{-1}\right)$ and *I*_[2]*k*_=1/((*I*_00_+1)^−1^+(*r*_0_*I*_00_+*r*_0_*Λ*_*k*_)^−1^).

Letting *R*_*k*_ denote the ratio *I*_[1]*k*_/*I*_[2]*k*_ and differentiating this with respect to *Λ*_*k*_ yields

$$\frac{dR_{k}}{d\Lambda_{k}}= \frac{ r_{0} I_{00}(a \Lambda_{k}^{2} + b \Lambda_{k} + c) } { (I_{00}+1)(1+r_{0})(I_{00}+\Lambda_{k})^{2} (\Lambda_{k} r_{0}+1)^{2} }. $$

with *a*=−(*r*_0_*I*_00_+2*r*_0_+1),*b*=2(*r*_0_−*I*_00_) and *c*=*I*_00_(*r*_0_+2)+1. Note that the derivative is defined for all *Λ*_*k*_≥0 as *I*_00_ and *r*_0_ are both positive. Setting the numerator to zero and solving the quadratic, we find that *R*_*k*_ has stationary points at *Λ*_*k*_=1 and −(*r*_0_*I*_00_+2*I*_00_+1)/(*r*_0_*I*_00_+2*r*_0_+1). The second of these is negative as *I*_00_ and *r*_0_ are positive, so that the only stationary point with *Λ*_*k*_≥0 is at *Λ*_*k*_=1 when *R*_*k*_=1

The second derivative of *R*_*k*_ with respect to *Λ*_*k*_ at *Λ*_*k*_=1 is equal to −2*r*_0_*I*_00_(*I*_00_+1)^−2^(*r*_0_+1)^−2^, and so is negative, confirming that the turning point is a maximum so that *R*_*k*_≤1, and hence *I*_[1]*k*_≤*I*_[2]*k*_, as stated.

## Appendix B: Type I error rate for Bayesian comparative trial with independent prior distributions

The requirement (5) that the error rate is controlled at level *α* in the two-paramter case can be stated as

11 $$ {\begin{aligned} Pr (Pr(\theta > 0 \mid \bar Y_{1k}, \bar Y_{0k}) \ge p_{k} \text{ some}\ k \le K; \mu_{1} = \mu_{0}) \le \alpha \text{ for all}\ \mu_{0}. \end{aligned}}  $$

We can rewrite the posterior distribuion (10) as $ \theta \mid \bar y_{1k}, \bar y_{0k}\sim N \left (M_{k}, I_{[2]k}^{-1} \right) $ with $ I_{[2]k}^{-1}= (I_{10}+I_{1k})^{-1} + (I_{00}+I_{0k})^{-1} $ and

12 $$  M_{k} = \frac{\mu_{10} I_{10}+ \bar y_{1k} I_{1k}}{I_{10}+I_{1k}} - \frac{\mu_{00} I_{00}+ \bar y_{0k} I_{0k}}{I_{00}+I_{0k}}.  $$

The posterior probability $pr(\theta > 0 \mid \bar y_{1k}, \bar y_{0k})$ is thus equal to $ 1 - \Phi \left (-M_{k} I_{[2]k}^{1/2} \right). $ This exceeds *p*_*k*_ whenever $ M_{k} \ge -\Phi ^{-1}(1-p_{k}) I_{[2]k}^{-1/2}. $

Hence in this case the stopping decision for the Bayesian sequential test depends on $\bar y_{1k}$ and $\bar y_{0k}$ via *M*_*k*_ and the frequentist operating characteristics for the Bayesian sequential test can be obtained from the joint distribution of *M*_1_,…,*M*_*K*_.

It follows from (12) and (1) that *M*_1_,…,*M*_*K*_ are multivariate normal with

$$E(M_{k}) = \frac{\mu_{10} I_{10}+ \mu_{1} I_{1k}}{I_{10}+I_{1k}} - \frac{\mu_{00} I_{00}+ \mu_{0} I_{0k}}{I_{00}+I_{0k}}. $$

When *μ*_1_=*μ*_0_, we have

$${\begin{aligned}E(M_{k}) = \frac{\mu_{10} I_{10}}{I_{10}+I_{1k}} - \frac{\mu_{00} I_{00}}{I_{00}+I_{0k}} + \mu_{0} \left (\frac{I_{1k}}{I_{10}+I_{1k}} - \frac{I_{0k}}{I_{00}+I_{0k}} \right). \end{aligned}} $$

If $\frac {I_{1k}}{I_{10}+I_{1k}} - \frac {I_{0k}}{I_{00}+I_{0k}} > 0$, we have *E*(*M*_*k*_)→*∞* as *μ*_0_→*∞*, and if $\frac {I_{1k}}{I_{10}+I_{1k}} - \frac {I_{0k}}{I_{00}+I_{0k}} < 0$, we have *E*(*M*_*k*_)→*∞* as *μ*_0_→−*∞*. In neither of these cases, then, is it possible to satisfy (11) for all values of *μ*_0_ other than in the trival case with *p*_1_=1, when stopping is impossible.

## Data Availability

Not applicable: no data or materials were used in this research.

## Abbreviations

CDAI: Crohn’s disease activity index
